# The Effect of Defect Size and Location in Roller Bearing Fault Detection: Experimental Insights for Vibration-Based Diagnosis

**DOI:** 10.3390/s25164917

**Published:** 2025-08-09

**Authors:** Haobin Wen, Khalid Almutairi, Jyoti K. Sinha, Long Zhang

**Affiliations:** 1 Dynamics Laboratory, The Department of Mechanical and Aerospace Engineering, School of Engineering, The University of Manchester, Manchester M13 9PL, UK; khalid.almutairi@manchester.ac.uk (K.A.); jyoti.sinha@manchester.ac.uk (J.K.S.); 2 Department of Mechanical Engineering, College of Engineering, University of Hafr Al Batin, P.O. Box 1803, Hafr Al Batin 31991, Saudi Arabia; 3 The Department of Electrical and Electronic Engineering, School of Engineering, The University of Manchester, Manchester M13 9PL, UK; long.zhang@manchester.ac.uk

**Keywords:** roller bearings, rotordynamics, defect size, fault detection, vibration-based condition monitoring

## Abstract

In rotating machines, any faults in anti-friction bearings occurring during operation can lead to failures that are unacceptable due to considerable downtime losses and maintenance costs. Hence, early fault detection is essential, and different vibration-based methods (VBMs) are explored to recognise incipient fault signatures. Based on rotordynamics, if a bearing defect causes metal-to-metal (MtM) impacts during shaft rotation, the impacts excite high-frequency resonance responses of the bearing assembly. The defect-related frequencies are modulated with the resonance responses and rely on signal demodulation for fault detection. However, the current study highlights that the bearing fault/faults may not be detected if the defect in a bearing is not causing MtM impacts nor exciting the high-frequency resonance of the bearing assembly. In a roller bearing, a localised defect may maintain persistent contact between rolling elements and raceways, thereby preventing the occurrence of impulse vibration responses. Due to contact persistence, such defects may not generate impact and may not be detected by existing VBMs, and the bearing could behave as healthy. This paper investigates such specific cases by exploring the relationship between roller-bearing defect characteristics and their potential to generate impact loads during operation. Using an experimental bearing rig, different roller and inner-race defects are presented while their fault characteristic frequencies remain undetected by the envelope analysis, fast Kurtogram, cyclic spectral coherence, and tensor decomposition methods. This study highlights the significance of both the dimension and location of defects within bearings on their detectability based on the rotordynamics concept. Further, simple roller-beam experiments are carried out to visualise and validate the reliability of the experimental observations made on the roller bearing dynamics.

## 1. Introduction

Anti-friction bearings are fundamental mechanical components that connect rotors and the supporting structure in rotating machines. Any bearing defects may cause malfunctions or even catastrophic failures, leading to considerable maintenance costs and downtime losses. Robust condition monitoring techniques play an important role in ensuring system reliability and sustainability.

For bearing fault detection, vibration condition monitoring is one of the most popular tools [1], where the concept of envelope analysis greatly helps to locate the exact fault(s) by identifying characteristic frequencies directly related to the defect(s) in different bearing components, i.e., the inner race, outer race, rolling elements and cage. Based on rotordynamics, when a defect occurs in an anti-friction bearing, the presence of the defect usually generates impact loadings due to metal-to-metal (MtM) contacts during shaft rotation. Such impact loadings usually excite decaying vibration responses at one or more high-frequency bands related to the resonance of the bearing assembly and housing. Depending on the defective components, one or multiple fault characteristic frequencies (FCFs) get amplitude modulated with the bearing resonance band(s) [2]. Since bearing resonance generally exhibits high-frequency broadband features in the acceleration spectrum, the modulation sidebands at the FCF interval are difficult to recognise. Hence, signal demodulation in the frequency band of the bearing resonance based on envelope analysis has been a cornerstone of many VBMs for bearing fault detection, as it effectively extracts the low-energy modulated components, i.e., bearing FCFs, by removing the carrier frequencies of bearing resonance [3]. The resulting envelope spectrum provides a clear indication of bearing faults by showing the FCF peaks if there is any defect. The concept of envelope analysis is significant, as it forms the foundation of numerous advanced diagnosis techniques, such as squared envelope spectrum or other improved envelope spectrum [4,5], optimal filtration band selection (e.g., fast Kurtogram [6] and Autogram [7]), and adaptive mode decomposition (e.g., variational mode decomposition [8,9], impulse mode decomposition, etc.), where the underlying rotordynamics for detecting fault impulses remain the same.

Despite the wide application of envelope analysis, the detection of early-stage faults remains challenging, as their weak signatures are easily submerged by operational and measurement noises. Hence, signal enhancement or source separation for early-stage faults has been targeted by recent research. For example, sparse representation and compressive sensing methods seek to isolate weak impulses from noisy vibration measurements using sparsity regularisation and dictionary learning [10]. Time-frequency analysis, such as short-time Fourier transform (STFT), continuous wavelet transform, and S-transform, provides a two-dimensional representation for weak signature enhancement instead of time-domain signals or frequency spectra. Furthermore, cyclostationary analysis enables frequency-frequency representations by quantifying the correlation between different frequency components, which detects the periodic statistics or modulation components in non-stationary signals, e.g., cyclic spectral coherence and the bi-frequency coherence map [11,12]. These representations pave the way for adaptive band decomposition techniques where fault detection is realised through blind source separation, such as independent component analysis, nonnegative matrix factorisation [13,14], and tensor decomposition [15,16]. These methods improve the signal processing approaches to clearly identify the FCF peaks in the case of faulty bearings, while the fundamental rotordynamic concept of the resonance responses generated by MtM impacts remains the same.

However, the sensitivity of vibration-based fault detection methods is influenced not only by defect severity and damage mode, but also by the bearing type. A large portion of existing VBMs are built upon experimental studies and public datasets on ball bearings (e.g., Prognostia and CWRU datasets [17,18]). Given the point contact between the bearing ball and raceway, a localised defect may invariably cause a complete loss of contact and lead to impact loads during rotation. These datasets are not used in the present study because they do not provide details of bearing defect sizes and their locations. In both ball and roller bearings, bearing faults generate modulating vibration responses as a result of MtM impacts. However, in ball bearings, the MtM impact-induced vibration responses are more likely to occur even for a relatively small fault size compared to roller bearings. For cylindrical roller bearings, the contact geometry between the roller and raceway can be regarded as a rectangular area, or ideally a line contact, which contributes to lower contact stresses and a higher load capacity. This fundamental difference may result in the VBMs designed for impulse fault detection in ball bearings not being directly applicable to roller bearing defects. Certain localised defects in roller bearings may not cause a complete loss of roller-raceway contact and the roller could pass the defect without creating any MtM impacts or machine malfunctions. In such cases, there are no impulsive vibration responses and the bearing behaves as healthy, rendering these defects undetected through classical VBMs.

To validate the above observations, this paper investigates typical roller bearing defects to explore the relationship between defect characteristics and their potential to generate impact loadings during operation. A roller bearing rig is developed to conduct bearing fault vibration experiments. A series of defects of varying dimensions are successively introduced on the roller and the inner-race to evaluate their detectability through envelope analysis and other advanced VBMs. The experimental results reveal the specific condition under which localised roller bearing defects do not produce MtM-detectable impact signatures. Further, simple roller-beam experiments are conducted to further visualise and validate the experimental observations made on the roller bearing dynamics. The presented work highlights that both defect size and location play significant roles in the fault detectability in roller bearings. Recognising these factors is essential for further development of reliable vibration-based bearing diagnostics. Moreover, the experimental observations contribute to critical insights on the detectability of roller bearing faults when applying envelope analysis, and then the comparative studies are also conducted against three other fault detection methods for validation, including fast Kurtogram [6], cyclic spectral coherence [11], and nonnegative tensor decomposition [15].

The paper is summarised as follows: Section 2 provides an overview of experimental settings, detailing the bearing test rig, the roller bearing, and various defects tested. Section 3 introduces the rotordynamic approach for bearing fault detection based on the envelope spectrum. The modal characteristics of the rig and the test bearing are first identified to enhance the reliability of envelope analysis. Section 4 presents the experimental results for the localised roller and inner-race defects that are undetected by envelope analysis, where fault impacts are not generated. A comparative study against three other advanced fault detection methods is elaborated in Section 5. Section 6 provides two typical inner-race defect cases causing metal-to-metal impact loadings. Furthermore, Section 7 presents the roller-beam experiments simulating healthy and defective inner race surfaces to visualise and validate the experimental findings on the roller bearing. Lastly, the concluding remark is made in Section 8.

## 2. Experimental Setup

### 2.1. Experimental Rig

**Figure 1:**
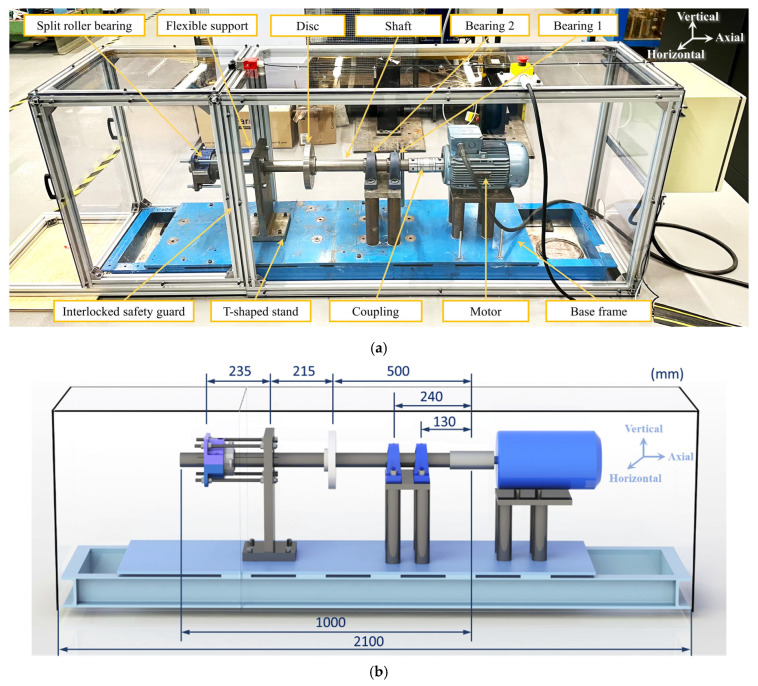
Experimental bearing rig. (**a**) Mechanical setup; (**b**) schematic diagram with dimensions.

The bearing rig used in this study was developed and installed in the Dynamics Lab of the University of Manchester. As shown in Figure 1a, the rotor-bearing system is installed on a thick steel base. The rig comprises a steel shaft of 50 mm diameter supported by two bearings and driven by an induction motor. A split roller bearing used for testing is mounted with a square flange housing at the end of the shaft, as shown in Figure 1a. The split roller bearing is used for the ease of creating different bearing defect scenarios and assembling on the rig for multiple vibration tests. The base frame is bolted to the lab floor with a viscoelastic layer in between for anti-vibration purposes. The speed of the rig can be controlled through a variable frequency drive. The detailed dimensions of the rig are provided in Figure 1b.

### 2.2. Roller Bearing for Testing

**Figure 2:**
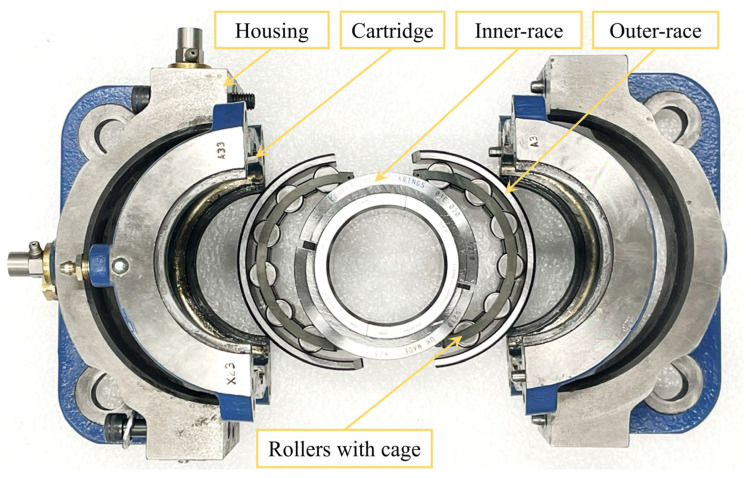
The components of the split roller bearing.

As shown in Figure 2, the roller bearing is splittable to the shaft and consists of a square flange housing, a cartridge, an inner-race with clamp rings, an outer-race, a cage, and rollers. Each of these bearing parts can be divided into two halves. The use of split bearings rather than solid bearings simplifies bearing installation and dismantlement between different defect experiments, minimising the influence on the shaft and the adjacent structures, thereby facilitating reliable and consistent experimental setups. Specifically, the detailed specifications of the split roller bearing are listed in Table 1.

### 2.3. Experimental Procedure with Seeded Bearing Defects

**Figure 3:**
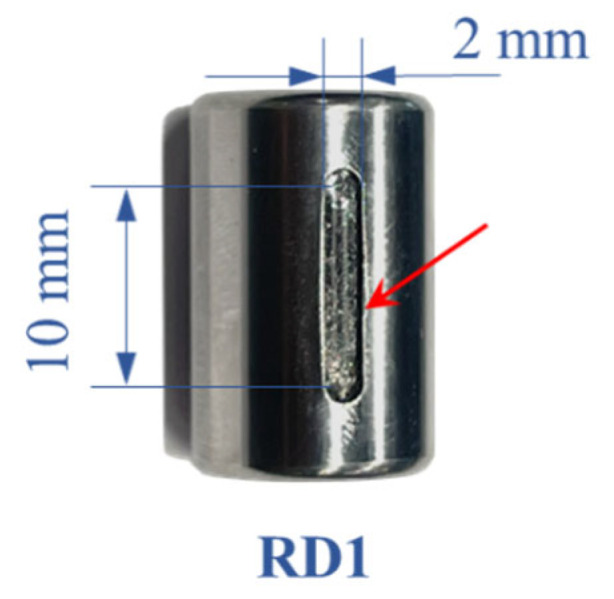
Roller defect RD1.

**Figure 4:**
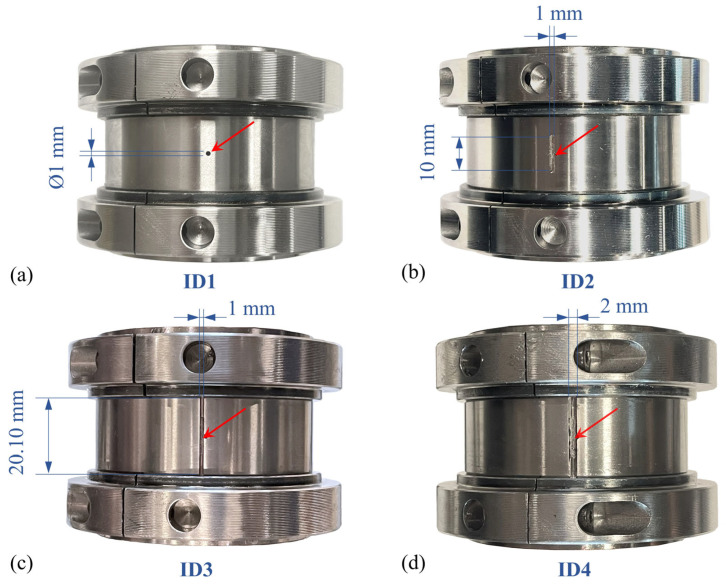
Inner-race defects. (**a**) ID1 point defect; (**b**) ID2 line defect; (**c**) ID3 line defect; (**d**) ID4 line defect.

The details of each bearing experiment, including the defect locations and dimensions, are specified in Table 2. The defect-free scenario, denoted as DF0, was conducted without any defects and considered healthy. As shown in Figure 3 and Figure 4, the roller defects (RD) and inner-race defects (ID) were generated via computer-numerical controlled (CNC) milling. Defect sizes are gradually increased for the experiments to show the change in dynamics, which also simulates the propagation of defect size with the machine operation. In each experiment, the bearing rig was operated under different rotation speeds with its self-load: 360 RPM (6 Hz), 720 RPM (12 Hz), 960 RPM (16 Hz), 1080 RPM (18 Hz), and 1320 RPM (22 Hz).

Two accelerometers are positioned on the square flange housing of the split bearing from vertical and horizontal directions using stud mounting to acquire bearing vibration signals. A proper sensor measurement range covering structural responses is a prerequisite for the reliability of vibration condition monitoring. Both accelerometers have a natural frequency above 50 kHz and an effective frequency range of up to 10 kHz, which are selected to cover the resonance bands of the split bearing assembly as fully as possible. The vibration signals were collected with a sampling rate of 25.6 kHz. For each defect scenario, vibration acceleration data were measured for 60 s during the steady state at the specified speed.

## 3. Results

### 3.1. Modal Test of The Laboratory Rig

**Figure 5:**
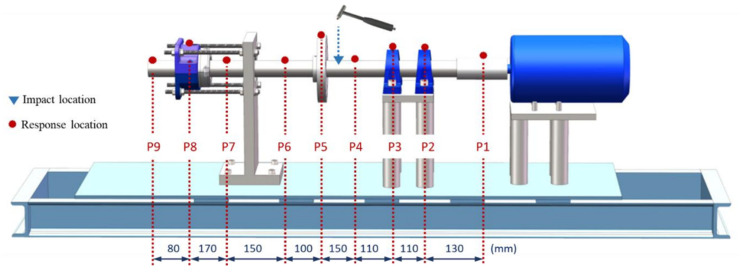
The measurement scheme used for the modal test of the rig (red dots represent locations of accelerometers in the vertical).

Modal tests were performed to identify the natural frequencies of the test rig for selecting appropriate operating speeds. As shown in Figure 5, an impact hammer with an in-built force transducer and a number of accelerometers along the rotor were used to identify the rig’s natural frequencies and mode shapes. The first five natural frequencies of the test rig based on the vertical measurements are summarised in Table 3.

### 3.2. Detection of Bearing Resonance Frequency Band

**Figure 6:**
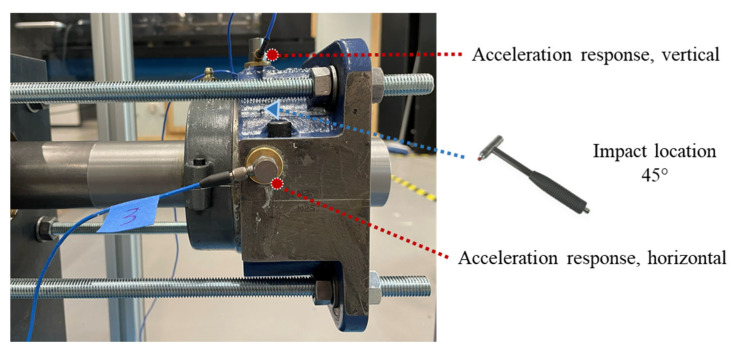
The hammer and accelerometer locations for identifying bearing resonance bands.

**Figure 7:**
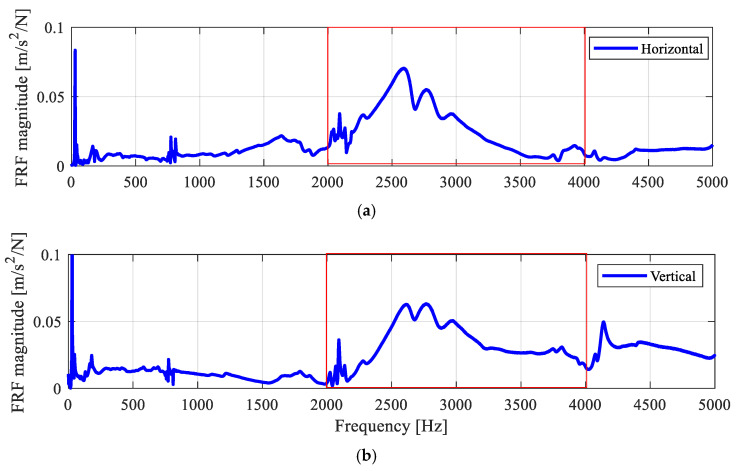
Magnitude spectra of the FRF (0–5 kHz), showing a common bearing resonance band from 2 to 4 kHz (as marked in the red box). (**a**) Horizontal direction; (**b**) vertical direction.

When defects develop in a bearing, fault characteristic frequencies are usually modulated with the resonance response of the bearing assembly. For demodulation, identifying the resonance band is essential for detecting bearing faults. Hence, a hammer test was carried out on the split bearing housing to detect the resonance frequency band of the test bearing assembly. As shown in Figure 6, the external excitation was given using the instrumented impact hammer, and the vibration responses were measured using two accelerometers mounted on the square flange housing from the horizontal and vertical directions. The measured inertance frequency response function (FRF) plots are shown in Figure 7. Based on the FRF, a common frequency band between 2 and 4 kHz observed in the FRF plots from both directions is identified as the bearing assembly resonance band.

### 3.3. Bearing Fault Detection Using Envelope Spectrum

When a bearing defect generates impact loadings, the bearing defect frequencies, or FCFs, get modulated with the resonance response of the bearing assembly. These metal-to-metal impacts are generated when bearing components (rollers, inner race, outer race, or cage) pass through the defect location during the shaft rotation. To detect FCF from the measured acceleration signals, amplitude demodulation is needed. Here, the envelope analysis is used for this purpose.

First, the measured acceleration signal is filtered using the passband corresponding to the bearing resonance band (2–4 kHz), as identified in Section 3.2. The filtration step removes the low-frequency components related to the rotor from the measured vibration signals and preserves only the bearing response [1,2]. The envelope of the filtered acceleration signal can be computed by:

(1) $$x\left(t\right)=\left|\;a\left(t\right)+\;jHilbert\left\{a\left(t\right)\right\}\right|,$$

where $x\left(t\right)$ represents the envelope of the filtered acceleration $a\left(t\right)$, *j* denotes the imaginary unit, and Hilbert{·} the Hilbert Transformation [2]. Subsequently, the envelope spectrum can be obtained by performing the fast Fourier transform (FFT). If there is a defect in the bearing, the envelope spectrum could efficiently show the spectral components matching the bearing FCFs and their harmonics.

Furthermore, it is suggested to compute the averaged amplitude envelope spectrum (AES) for an enhanced spectral representation by reducing random measurement noises. The AES is computed as in Equation (2) based on the concept of power spectral density [2],

(2)  XAES(fk)=(EXfkX*fk)12=∑i=1MXi(fk)Xi*(fk)M 12

where $X_{AES}(f_{k})$ denotes an amplitude envelope spectrum at the frequency $f_{k}$, based on M averaging, $X_{i}(f_{k})$ and $X_{i}^{*}\left(f_{k}\right),$ respectively, represent the complex envelope spectrum and its conjugate of the *i*-th envelope segment ($x_{i}$). Each data segment consists of *N* FFT points ($N=2^{m}$). The frequency index is computed as $f_{k}\in [0,\frac{f_{s}}{2}]$ ($k=0, 1,\;\ldots ,\frac{N}{2}-1$), where $f_{s}$ is the sampling frequency.

## 4. Analysis of Bearing Defects Without Generating Impact Loads

A few damages are created in the roller and inner race such that they should not create any metal-to-metal impact loadings, irrespective of shaft rotating speeds and loads. Due to the fault size, geometry and the remaining contact in the bearing, these faults are not going to excite the bearing resonance, and thus, no modulation of the FCFs will be formed in the bearing acceleration response. As a result, VBM is not going to detect these cases as faulty, as demonstrated below.

The measured vibration signals are analysed to compute the AES, as discussed in Section 3. The measured vibration data are initially filtered using the bandpass filter of 2 to 4 kHz (the bearing resonance frequency band). The envelope signals are then extracted using Hilbert Transformation as per Equation (1). The AES is computed using $N=2^{19}$ and a total of M=10 averages, with the frequency resolution ($\frac{f_{s}}{N}$) at 0.0488 Hz selected for distinguishing between informative frequencies, such as bearing fault frequencies and the higher harmonics of the shaft speed.

In this section, the vibration acceleration data collected at the synchronous shaft speed of 709 RPM (11.82 Hz) are presented. The motor drive speed is set at 720 RPM (12 Hz) during experiments, with the actual speed found to be 709 RPM. Based on the dimensions of the roller bearing (see Table 1) and the experimental shaft speed, the FCFs for different bearing components are computed as per the corresponding rotational kinematic formulae [2], which are listed in Table 4.

### 4.1. Defect-Free Case-Healthy Condition

**Figure 8:**
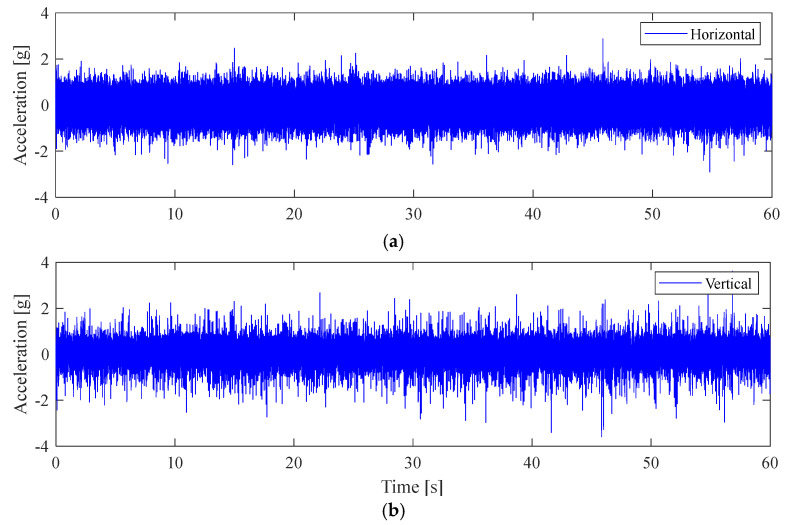
Measured acceleration signals on the bearing with no defect (DF0). (**a**) Horizontal; (**b**) vertical.

**Figure 9:**
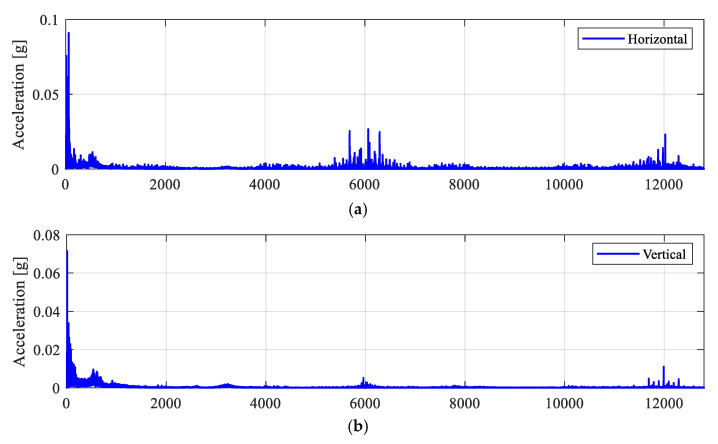

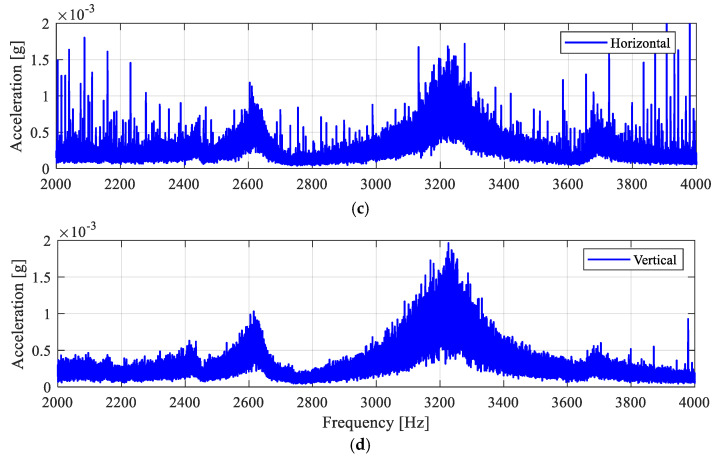
Measured acceleration spectra for the DF0 case. (**a**) Horizontal; (**b**) vertical; (**c**) horizontal (zoomed-in view between 2 and 4 kHz); (**d**) vertical (zoomed-in view between 2 and 4 kHz).

The experiments start with the defect-free (DF0) roller bearing to collect vibration data under healthy conditions. The measured acceleration signals from the horizontal and the vertical directions are shown in Figure 8. Figure 9a,b present their corresponding amplitude spectra. The zoomed-in views of the spectra in the bearing resonance frequency band of 2–4 kHz are also shown in Figure 9c,d. The envelope spectra (AES) are then computed as per Section 3 and Section 4.

**Figure 10:**
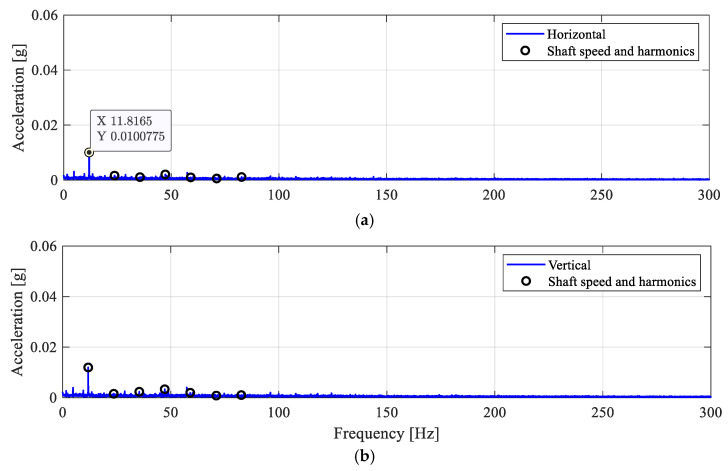
Envelope spectra for the DF0 case. (**a**) Horizontal; (**b**) vertical.

The corresponding AES are shown in Figure 10. As expected, since the roller bearing operates under the healthy condition, the AES from the horizontal and vertical directions are only showing a dominant frequency peak at the synchronous shaft speed, as marked by black circles. For both directions, the acceleration amplitudes of the ‘1×’ shaft speed components are approximately 0.01 g (*n*× is used to denote the *n*-th harmonics of a frequency component).

### 4.2. Roller Defect Case (RD1)

**Figure 11:**
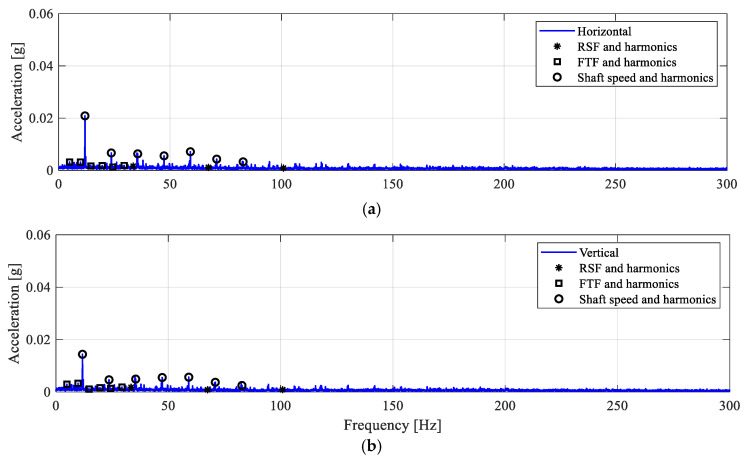
Envelope spectra for the RD1 case. (**a**) Horizontal; (**b**) Vertical.

**Figure 12:**
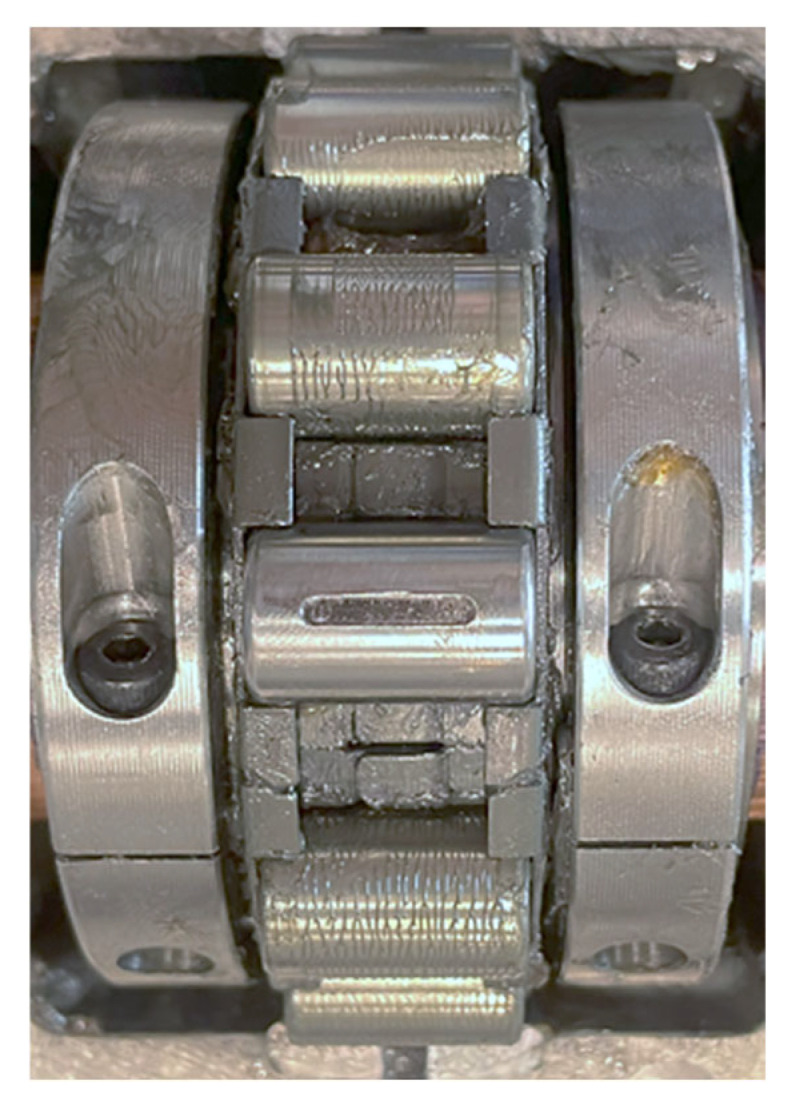
An illustration of roller-race interaction for the RD1 case. The roller with the RD1 defect could smoothly pass the cage and both bearing races.

As shown in Figure 3, roller defect RD1 is seeded with the dimensions specified in Table 2. A line defect (straight slot) in the middle of the roller surface is developed such that the length of the slot is smaller than the inner and outer race width. Hence, a partial loss of roller-raceway contact is introduced during bearing operation. The computed envelope spectra are shown in Figure 11. It is seen that the AES are only dominated by the shaft speed component at 1× with a few higher harmonics of smaller amplitudes. No frequency peak at the RSF is observed. This implies that the roller defect RD1 is not detected since no metal-to-metal impact loading is generated by RD1. It also indicates that the defect does not result in bearing resonance and that no roller malfunction is observed. To enhance clarity, a geometric illustration of the contact between the faulty roller and the bearing races is presented in Figure 12. The figure clearly demonstrates that, as the RD1 is centred at the axial length of the roller, the remaining contact between the faulty roller and the raceway supports the roller to smoothly pass over the bearing races without metal-to-metal collision or impact loadings.

### 4.3. Inner-Race Defect Cases

**Figure 13:**
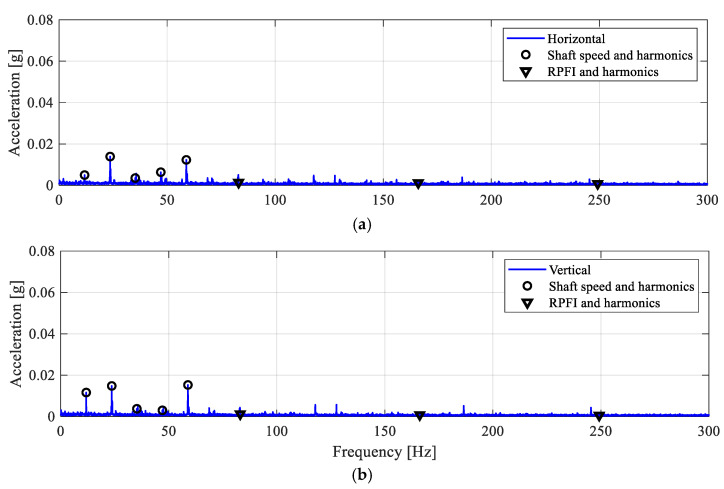
Envelope spectra for the ID1 case. (**a**) Horizontal; (**b**) vertical.

For inner-race defect cases (see Table 2 and Figure 4), ID1 is a point defect of 1 mm diameter on the inner-race surface and centred on its axial width. Figure 13 shows the envelope spectra of the filtered vibrations from both accelerometers. It is seen that both envelope spectra only show low-amplitude peaks at the shaft speed and its harmonics. Whereas the inner-race defect frequencies, RPFI harmonics (see Table 4), are not observed. Since the inner-race fault frequencies are not observed from the envelope spectra, it can be concluded that ID1 does not interrupt smooth contact between rollers and inner-race, nor generate impacts during the bearing operation. Hence, the inner-race defect ID1 is not detected.

**Figure 14:**
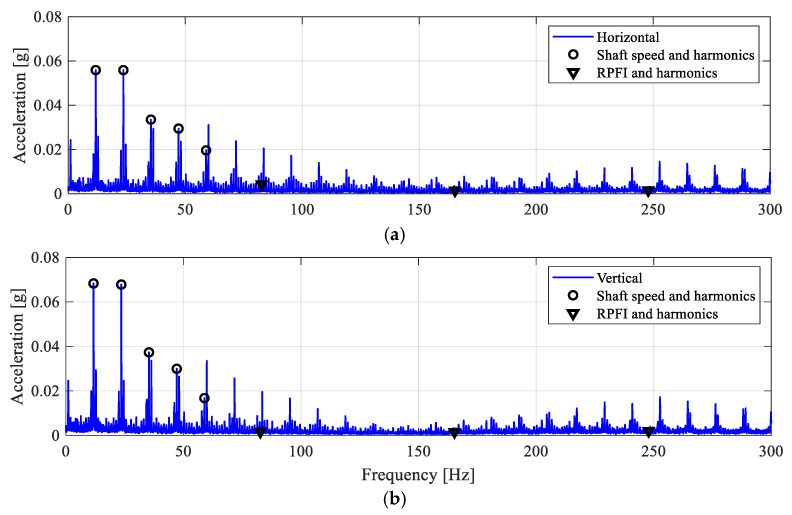
Envelope spectra for the ID2 case. (**a**) Horizontal; (**b**) vertical.

Similarly, the vibration data of the inner-race defect ID2 (see Table 2 and Figure 4) are also analysed. The defect ID1 is further widened at the centre of the inner race width to create the inner-race surface defect ID2. The size of the straight slot defect is 1 mm wide and 11 mm long along the inner race width. The envelope spectra of the filtered vibration signals are shown in Figure 14. It can be seen that only the frequency peaks at the shaft speed and its higher harmonics are observed but with higher amplitudes as compared to the ID1 case. This may be a result of the residual misalignment in the rotor shaft. However, no distinctive frequency peaks are found matching the inner-race defect frequency, RPFI. Once again, it is clear that the defect size of ID2 (larger than ID1) and its location on the inner race allow the rollers to roll over the inner race smoothly. For this reason, the inner-race defect ID2 does not generate impact loadings during bearing operation. Since impact fault modulation is not established, ID2 is not detected by resonance demodulation.

## 5. Comparative Study Using Different Detection Methods

### 5.1. Machine Speed-720RPM

To strengthen the observations derived from envelope analysis in Section 4, the measured vibration data from the non-impulsive defect cases are further analysed using different methodologies. First, as a well-recognised benchmark for optimal band selection, the fast Kurtogram (FK) utilises a filter-bank tree to decompose signals and identifies the most informative frequency band based on the maximum filtered kurtosis [6]. Using the high-kurtosis filtration band, a squared envelope spectrum (SES) is derived to enhance the visibility of bearing fault characteristics and facilitate more effective fault detection.

**Figure 15:**
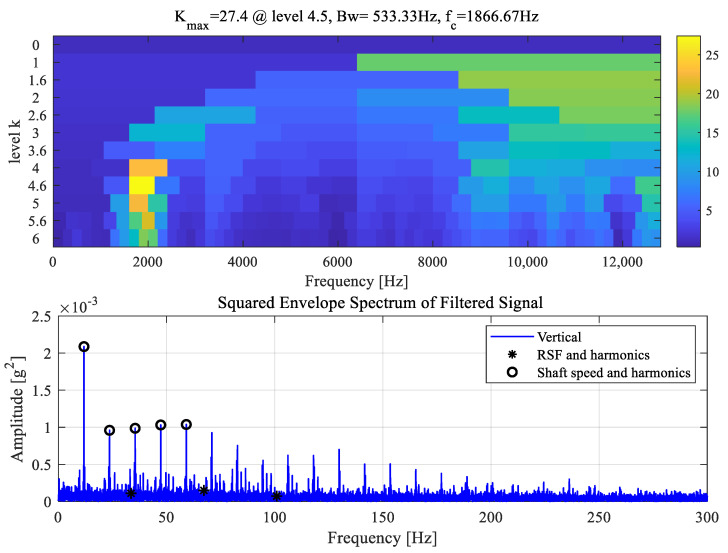
Fast Kurtogram and SES for the roller defect case RD1.

Figure 15 shows the results of FK using the vertical acceleration signal in the roller-defect experiment (RD1). It can be seen that the ‘optimal’ band is identified at the centre frequency of 1866.67 Hz with the maximum filtered kurtosis. The corresponding squared envelope spectrum (SES) is computed based on the squared envelope of the filtered signal using the passband from 1600 to 2133 Hz. It is shown that the SES is dominated by the apparent shaft speed component and its higher harmonics, while the roller defect frequency, RSF, is not identified. Therefore, the roller defect RD1 is not detected via FK.

Due to the shorter length of RD1 than the axial roller length, the defective roller can be supported by the remaining contact to operate smoothly without metal-to-metal impacts. Albeit, the partial loss of the roller-race contact in RD1 results in a variation of local contact stiffness, which may cause vibration modulation even without fault-induced impulses. In such cases, fault detection methods based on envelope analysis can be limited since impact-induced amplitude modulations are not formed.

Thus, cyclostationary analysis is further introduced to identify the modulation pattern in a cyclostationary signal, whose high-order statistics vary periodically with time [19]. Specifically, the cyclic spectral coherence (SC) method [11] is applied for further comparison, in which a bi-frequency map is formulated to indicate the correlation between different frequency components, i.e., the spectral frequency representing the carrier frequency component and the cyclic frequency (α) representing the periodic or modulation component. Then, an enhanced envelope spectrum (EES) is derived based on the average spectral coherence over the spectral frequency axis to identify the principal modulation components [11,20].

**Figure 16:**
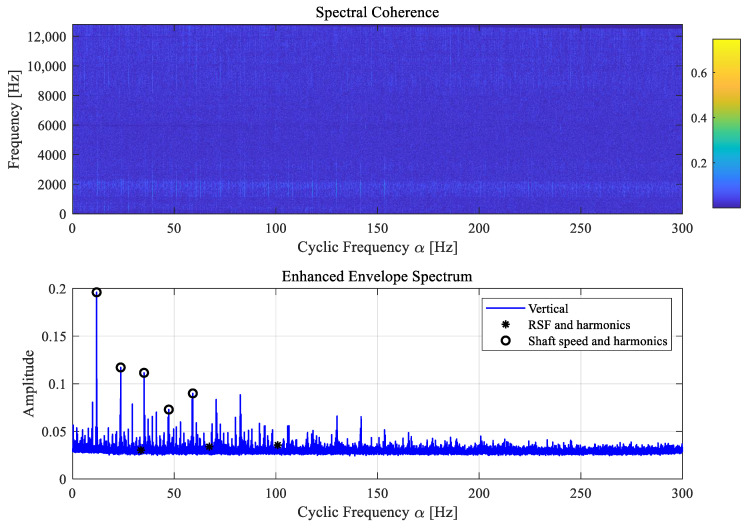
The spectral coherence map and the EES for the roller defect case RD1.

From Figure 16, repetitive vertical spectral lines can be observed from the SC map, while the corresponding EES indicates that these dominant modulation frequencies are indeed the shaft speed harmonics. Besides, the roller defect frequencies at RSF are not observed. Hence, the result indicates that the roller defect RD1 is not detected using cyclic spectral coherence. As the modulation frequency induced by RD1 is not detected, it can be inferred that the variation in contact stiffness is likely minor relative to the bearing stiffness.

For further comparisons, a recent adaptive frequency band decomposition method based on non-negative tensor decomposition (NTD) of time-frequency (TF) spectrograms is leveraged [15]. Without additional modal testing, NTD provides an adaptive multi-way data fusion method to extract inherent temporal components and their corresponding spectral bands from vibration measurements. Here, the spectrograms of the acceleration signals from both horizontal and vertical channels are first computed based on the short-time Fourier transform (STFT). The three-way TF tensor is formed by stacking multiple spectrogram segments. Based on nonnegative Tucker decomposition, the TF tensor is factorised into the spectral factor, temporal factor, segment-wise factor, and the core tensor for revealing the correlation across multiple physical dimensionalities. By decomposition, an improved cross-spectrum (ICS) can be obtained using the dominant temporal component for bearing fault detection.

**Figure 17:**
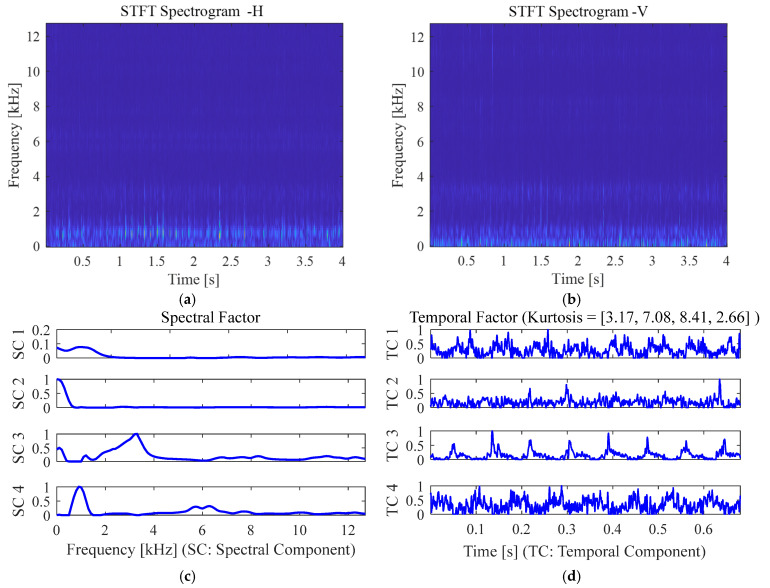
The spectrograms of the measured acceleration signals from RD1 and the decomposed factors via NTD using the factorisation rank of (4,4,2). (**a**,**b**) STFT spectrograms of the vibration signals from the horizontal and vertical channels; (**c**) spectral factor; (**d**) temporal factor.

**Figure 18:**
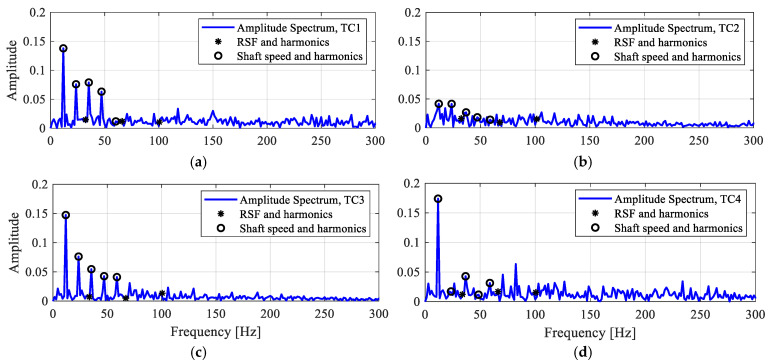
The ICS of four dominating temporal components for the roller defect case RD1. (**a**) Amplitude spectrum of TC1; (**b**) amplitude spectrum of TC2; (**c**) amplitude spectrum of TC3; (**d**) amplitude spectrum of TC4.

Figure 17a,b show the STFT spectrograms of the two-channel vibration acceleration signals from the roller defect case RD1. The decomposed spectral factor with four dominant spectral bands is presented in Figure 17c. The principal temporal components (TC) are extracted in Figure 17d, where TC 3 has the maximum kurtosis value. The improved cross spectra are computed using the fast Fourier transform of the temporal components, as shown in Figure 18. It can be seen that based on multi-sensor data fusion, the ICS of most temporal components, including TC1, TC3, and TC4, are still dominated by the shaft speed harmonics, while the roller defect frequency, RSF, remains undetected.

Apart from the roller defect case, the inner-race defects (ID1 and ID2) are also examined. Table 5 summarises the fault detection performance of the different methodologies. The result clearly indicates that the selected methods are in a consistent agreement: no bearing fault characteristic frequencies are observed in the spectral results for the roller defect (RD1) and two inner-race defects (ID1 and ID2).

### 5.2. Comparisons at Other Machine Speeds

The vibration data are also collected under different operating conditions, as listed in Section 2.3. All the data are analysed using the methods mentioned above. The observations are exactly the same as in Table 5, irrespective of the shaft speeds. Therefore, the analysed roller defect and inner-race defects do not induce impacts or cause resonance in the roller bearing assembly. Since impact-induced amplitude modulation is not generated, the fault characteristic frequencies cannot be effectively detected by envelope analysis and other examined vibration-based methods for bearing fault detection. This means that even though the bearing is subjected to different rotor unbalance forces due to low or high speeds, the dynamic behaviour remains unchanged in terms of no MtM impacts.

## 6. Analysis of Roller Bearing Defects Generating Impact Loads

The inner-race defect ID3 (see Table 2 and Figure 4) is further extended to a longer line defect up to the width (both edges) of the inner-race surface (1 mm width and depth and 20.10 mm length). The defect size (length) has been increased such that the bearing experiences a repetitive and complete loss of roller-raceway contact during its operation, causing metal-to-metal collisions as the roller drops onto the inner-race defect.

**Figure 19:**
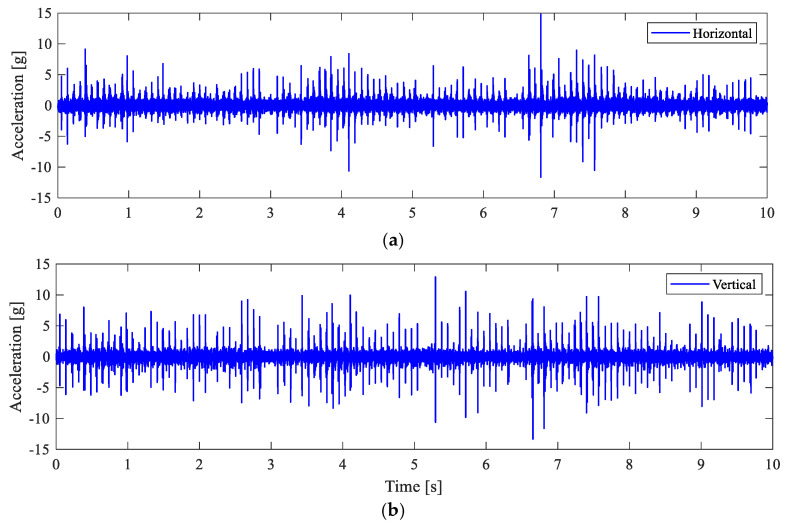
Measured acceleration signals (a zoomed-in plot of 10 s). (**a**) Horizontal; (**b**) vertical.

**Figure 20:**
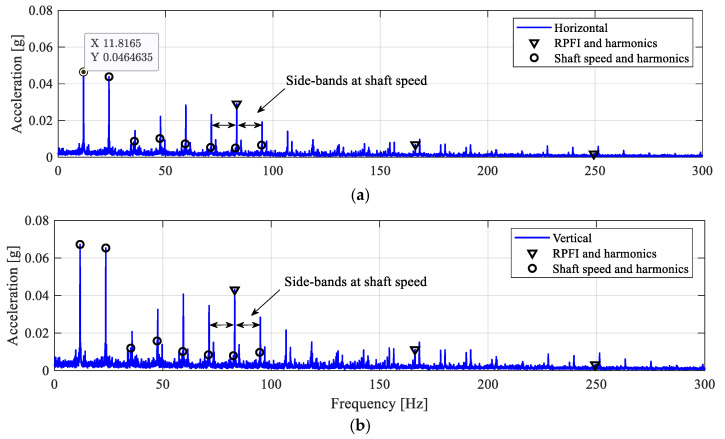
Envelope spectra for the ID3 case. (**a**) Horizontal; (**b**) vertical.

The measured acceleration signals are shown in Figure 19, with noticeable impulsive behaviours. The envelope spectra of the filtered acceleration signals in Figure 20 clearly show the presence of the RPFI frequency peak in both directions, along with the 1× and its higher harmonics. Hence, this observation indicates that the bearing malfunction leads to impact loadings, which confirms the existence of an inner-race fault in the roller bearing.

**Figure 21:**
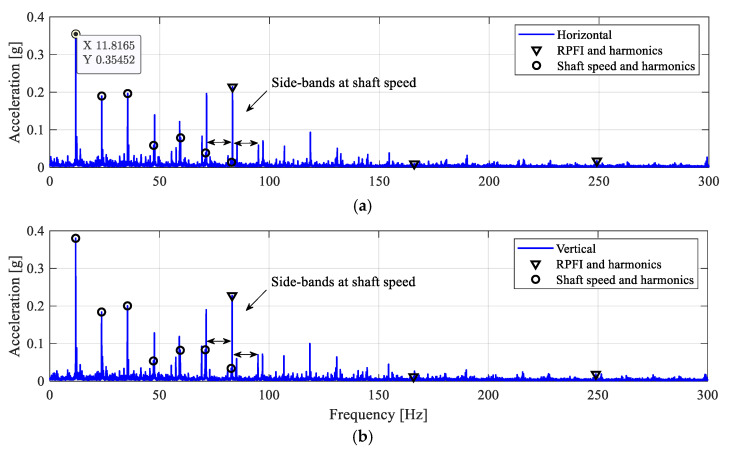
Envelope spectra for the ID4 case. (**a**) Horizontal; (**b**) vertical.

Next, the inner-race defect ID4 (see Table 2 and Figure 4) is a wider line defect extending from ID3 to a 2 mm-width notch over the inner-race surface. The envelope spectra of the filtered acceleration signals are shown in Figure 21. The frequency peaks at the inner-race defect frequency, RPFI, can be clearly identified from both the horizontal and vertical directions. Distinctively, the acceleration amplitudes at RPFI for the ID4 case are above 0.2 g in both measurement directions, which are much higher than the ID3 case with a smaller defect size. This observation again confirms the presence of impact loadings and the inner-race defect in the roller bearing.

## 7. Further Validation: Roller-Beam Experiments

**Figure 22:**
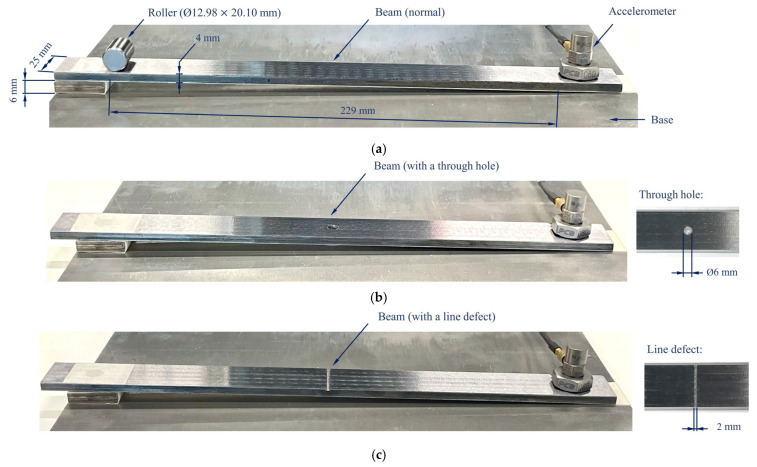
Three different roller-beam setups for testing defect-induced vibrations. (**a**) Normal beam; (**b**) beam with a centred through hole; (**c**) beam with a line defect.

To visualise and validate the observations made on the roller bearing dynamics, a simple experimental setup of a steel beam and a bearing roller, as shown in Figure 22a, was used. The experiment setup assumes that the bearing inner-race was open as a straight beam. The vibration experiments were conducted by rolling the cylindrical roller of the test bearing over the beam with healthy and different defective surfaces. Three different experiments were conducted, which are illustrated in Figure 22. The left end of the steel beam was lifted by a steel block of 6 mm height to allow the roller to roll naturally and freely during the experiments. To simulate different contact geometries and metal-to-metal impact, three conditions were simulated, namely a healthy beam surface (no inner race defect), a Ø6 mm hole on the beam surface (defect on the inner race), and a 2 mm-width line defect (wider defect on the inner race). An identical surface finish (polished using a grinding machine tool) for the steel beams was maintained during the experiments.

**Figure 23:**
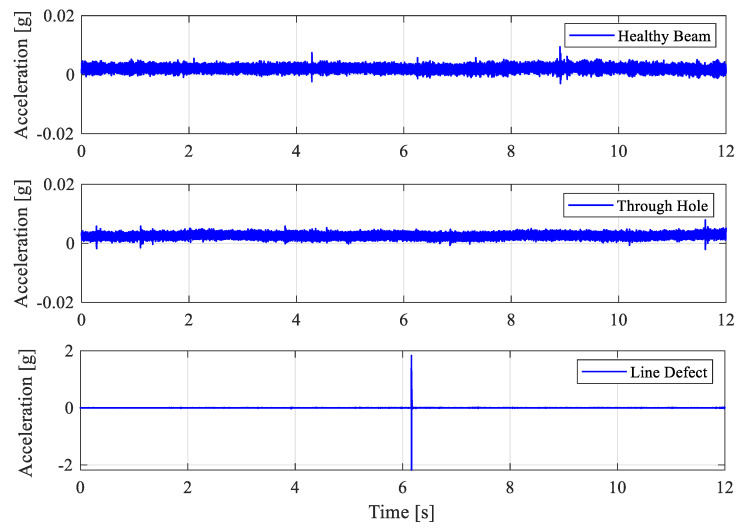
The measured vibration acceleration signals in three experiments, namely no defect, a small hole defect, and a wider line defect.

The roller was released from the same starting location as shown in Figure 22 for all 3 experiments. The accelerometer was mounted on the beam at the lower end of the beam for measuring the vibration response excited by the roller as it travels along the beam. Each experiment was repeated 3–4 times to confirm the repeatability of the dynamics. Typical measured vibration acceleration signals are shown in Figure 23.

For the healthy beam surface (simulating no inner race defect), the vibration signal exhibits a stationary vibration response pattern at low acceleration amplitude below 0.01 g. The measured vibration signal shown in Figure 23 for the beam with a small hole (simulation of a small defect in the inner race) is observed to be nearly the same as the healthy beam surface case. This confirms that roller-beam contact continues to support the roller and allow it to pass over the defect hole smoothly without generating metal-to-metal impact collisions. However, the measured acceleration signal in the line defect scenario (simulating a wider inner race defect) shows a prominent impulse around the 6th second with an amplitude of approximately 2 g, as the roller runs in and out of the defect area. The line defect on the beam causes a sudden and complete loss of support for the roller, ending up with the impulse vibration response excited by the metal-to-metal impact.

The simple experiments confirm the observations made in the roller bearing dynamics. Therefore, if any defect in the bearing does not generate an impulse vibration response, then it is difficult to detect bearing defects using the VBMs.

## 8. Concluding Remarks

This study investigated the detectability of roller bearing faults using VBMs through a few typical experimental examples. The study primarily utilises rotordynamics behaviours and focuses on the influence of the defect size and location on bearing fault detection and their detectability. The VBMs generally identify the presence of the bearing fault(s), if the defect size and its location can generate MtM impact loads within the bearing during shaft rotations. These impact loads excite resonances of the bearing assembly and lead to the modulation between FCFs and bearing resonance responses, hence indicating the bearing malfunction.

In this study, a few defects are deliberately created in the roller and the inner race such that these defects should not cause impact loads and impulsive vibration responses of the bearing. Bearing vibration experiments are conducted at different rotating speeds to observe the fault detection performance of different VBMs and the underlying rotordynamic concept. The presented cases clearly indicate that the VBMs (envelope analysis, fast Kurtogram, cyclic spectral coherence, and nonnegative tensor decomposition methods) do not detect the bearing fault if the defect does not generate any MtM impacts, irrespective of the shaft speeds. These defects are centred on the roller or raceway surface with localised sizes that do not lead to a complete loss in roller-raceway contact. In such cases, they do not lead to bearing malfunctions, and smooth machine operation can be maintained. On the other hand, in the larger inner-race defect cases, the defect causing a complete loss of roller-raceway contact excites MtM impact loads and generates impulse vibration responses. The VBMs could detect such defects easily and reliably. This simply means that if there are no impulsive vibration responses due to the defect-induced MtM impact loads, then the bearing defect is not detected by the VBMs. As VBMs are grounded in the principles of machine rotordynamics, early bearing fault detection using VBMs is generally possible only if the fault initiates MtM impacts.

The simple roller-beam experiments also validate the observations made on the dynamics of the roller bearing. In summary, this work provides critical insights into the effect of defect size and location on fault detectability for roller bearings when using vibration-based methods. By demonstrating the conditions under which bearing defects can and cannot be detected, the study offers valuable guidance for both industry applications and ongoing research in this field. These findings also contribute to a deeper understanding of bearing fault diagnostics based on rotordynamic behaviours and provide a future scope for further research studies using different sensors other than vibration sensors.

## Figures and Tables

**Table 1:** Specifications of the split roller bearing.

Parameter	Value	Parameter	Value
Bore diameter	50.00 mm	Contact angle	0°
Outside diameter	98.42 mm	Mass	1.5 kg
Pitch diameter	76.14 mm	Radial dynamic load rating	95.00 kN
Outer diameter of inner race	63.16 mm	Radial static load rating	105.00 kN
Roller diameter	12.98 mm	Axial load rating	3.80 kN
Number of rollers	12	Speed limit	4630 RPM

**Table 2:** A summary of seeded roller-bearing defects.

Defect Location	Notation	Defect Shape	Defect Dimension (*W*: Width, *L*: Length, *D*: Depth)
None	DF0	-	-
Roller	RD1	Line defect	2 × 12 × 1 mm (W × L × D)
Inner race	ID1	Circle point defect	1 mm diameter, 1 mm depth
	ID2	Line defect	1 × 11 × 1 mm (W × L × D)
	ID3	Line defect (Edge-to-edge)	1 × 20.10 × 1 mm (W × L × D)
	ID4	Line defect (Edge-to-edge)	2 × 20.10 × 1 mm (W × L × D)

**Table 3:** First five natural frequencies of the bearing rig.

	1st	2nd	3rd	4th	5th
Natural frequency (Hz)	28.91	48.05	59.77	76.34	129.69

**Table 4:** Bearing fault characteristic frequencies based on the experimental shaft speed.

Measured Shaft Rotation Speed	Fundamental Train Frequency (FTF)	Roller Spin Frequency (RSF)	Roller Pass Frequency Outer (RPFO)	Roller Pass Frequency Inner (RPFI)
11.82 Hz	4.90 Hz	33.66 Hz	58.83 Hz	83.01 Hz

**Table 5:** Fault detection results using other advanced vibration-based methods.

Defect Cases	Roller Defect	Inner-Race Defect	
	RD1	ID1	ID2
Method 1: Amplitude Envelope Analysis (AES)	N *	N	N
Method 2: Fast Kurtogram (SES)	N	N	N
Method 3: Cyclic Spectral Coherence (EES)	N	N	N
Method 4: NTD-based Sensor Fusion (ICS)	N	N	N

* ‘N’: Bearing fault characteristic frequencies (FCFs) are not observed from the spectral results.

## Data Availability

The data presented in this study are available upon request from the corresponding author.
